# Long-Term Results of Percutaneous Balloon Aortic Valvuloplasty in Children With Aortic Stenosis: A Single-Center Experience

**DOI:** 10.7759/cureus.64547

**Published:** 2024-07-15

**Authors:** Eser Doğan, Ertürk Levent

**Affiliations:** 1 Pediatric Cardiology, Ege University, Izmir, TUR

**Keywords:** heart failure, percutan, balloon angioplasty, pediatric cardiologist, critical aortic stenosis

## Abstract

Purpose: Congenital aortic stenosis is a common pathology in the childhood age group and its clinical spectrum varies between asymptomatic and severe heart failure. In our study, we planned to evaluate the long-term results of patients who underwent balloon aortic valvuloplasty (BAV) due to critical aortic valve stenosis in our clinic.

Materials and methods: Patients aged 0-18 years who underwent aortic balloon valvuloplasty due to aortic stenosis in our clinic between January 2002 and January 2022 were retrospectively evaluated.

Results: Among the 48 patients who underwent balloon valvuloplasty due to aortic stenosis, 13 (27%) were female, and 35 (73%) were male. The median age at the time of the procedure was 27.5 months (IQR: 4-96), the median weight was 9.9 kg (IQR: 5.40-29.50), and the median height was 79 cm (IQR: 54-133). The median follow-up duration was 93.5 months (IQR: 38-132). Angiographic assessments in all patients revealed a median left ventricular pressure of 160 mmHg (IQR: 140-200) and a median pressure gradient between the left ventricle and the aorta of 60 mmHg (IQR: 42-80). The median balloon diameter used was 10 mm (IQR: 8-12). Post-procedural measurements showed a median mean gradient of 30 mmHg (IQR: 20-35) between the left ventricle and the aorta. The procedure was successful in 45 (93.5%) patients. During follow-up, 11 patients required surgical intervention. Ross procedure was performed in five patients, homograft in five patients, and mechanical valve implantation in one patient. Risk factors for the need for surgical intervention were evaluated in detail. During the follow-up, the risk factor for intervention was determined to be aortic insufficiency.

Conclusion: Aortic valve balloon valvuloplasty is a safe and successful treatment method for critical aortic stenosis. It should be the first choice of treatment option in suitable patients.

## Introduction

Congenital aortic valve stenosis is a relatively common cardiac anomaly, constituting 2-7% of all congenital diseases [1-3]. The disease presents a spectrum of symptoms from clinically asymptomatic patients to severe heart failure symptoms, with a mortality rate of up to 15% in untreated cases [2]. Balloon valvuloplasty is the primary option for aortic stenosis. Its advantages over surgical treatment include being less invasive, shorter hospitalization periods, and often not requiring intensive care unit (ICU) admission.

The success of aortic balloon valvuloplasty depends not only on the anatomy of the aortic valve but also on the spectrum of associated left heart anomalies, such as borderline left ventricle, endocardial fibroelastosis, and mitral valve abnormalities, which can affect prognosis [4-6]. Following aortic balloon valvuloplasty, there is often a need for reintervention either angiographically or surgically during clinical follow-up. It has been observed that in patients undergoing balloon valvuloplasty due to congenital aortic valve stenosis, a high degree of post-procedural aortic valve insufficiency and/or residual gradients ≥35 mmHg increases the risk of reintervention during mid-term follow-up [7].

Considering the studies in the literature, the rates of reintervention after aortic balloon valvuloplasty are high. Our aim in this study is to identify factors predicting the need for reintervention during long-term follow-up in patients who underwent transcatheter aortic balloon valvuloplasty at Ege University Pediatric Cardiology Department, a tertiary care center.

## Materials and methods

Study design and population

Patients aged 0-18 years who underwent aortic balloon valvuloplasty at the Department of Pediatric Cardiology, Ege University Faculty of Medicine Hospital, retrospectively between January 2002 and January 2022, were included in the study. Patients with complex cardiac anomalies accompanying aortic stenosis who underwent surgical treatment for these anomalies were excluded from the study. The presence of patent ductus arteriosus (PDA) or patent foramen ovale in newborns was not considered hemodynamically significant. Patients with incomplete data were also excluded from the study. Written consent was obtained from patients and caregivers. Ethical approval was obtained from the Ethics Committee of Ege University Faculty of Medicine (decision no: 23-11.2T/26; decision date: 30.11.2023).

Hemodynamic assessment

Transthoracic echocardiographic examinations of the cases were performed using the Vivid e9 echocardiography device (GE, Vingmed Ultrasound, Norway). Two-dimensional, M-mode, color flow Doppler, pulsed Doppler, and continuous wave Doppler echocardiography were used in all patients. Preoperative evaluations were performed before surgical operation or balloon valvuloplasty. The number of cusps on the aortic valve was evaluated using two-dimensional echocardiography from the parasternal short-axis view. Continuous wave Doppler was used via the suprasternal notch or right parasternal windows to record the maximum peak systolic instantaneous gradient (TSEG=4xmaximum velocity^2) on the aortic valve. Congenital aortic valve stenosis was defined as an inborn abnormality of the aortic valve with a flow velocity of at least 3 m/second across it and/or significantly decreased aortic valve area on echocardiography.

Valvular insufficiencies at the time of admission were recorded in patients. Changes in the severity of insufficiency during follow-up were examined. Aortic valve insufficiency was classified according to echocardiographic assessment as follows: absent, mild, moderate, and severe. Absent: no insufficiency present. Mild: mild broad insufficiency flow limited by systolic flow convergence velocity (SFCV) below the aortic valve without accompanying ventricular dilation. Moderate: insufficient flow extending into the left ventricle. The left ventricle may be mildly dilated. Retrograde flow is seen within the descending aorta. Severe: wide insufficiency flow extending deep into the left ventricle. The left ventricle is significantly dilated. Holodiastolic retrograde flow is seen within the descending aorta.

Balloon angioplasty procedure

Transcatheter balloon aortic valvuloplasty (BAV) was indicated in accordance with contemporary guidelines [6]. Cases with a pressure gradient of 50 mmHg or higher between the left ventricle and aorta in pressure measurements, cases with a pressure gradient lower than 50 mmHg but with ST-T wave changes on electrocardiography, unexplained dizziness and/or syncope, and cases with significant chest pain underwent BAV. All cases underwent intervention via retrograde femoral artery access and antegrade femoral and umbilical vein access under ketamine and midazolam anesthesia. Aortic root injection was performed in a 30-degree left anterior oblique position using a "Pigtail" catheter to assess the aortic root. Cases with significant aortic insufficiency were not subjected to BAV. Measurement of the aortic valve annulus diameter was performed by considering both repeated echocardiographic measurements the day before the procedure and measurements obtained from aortic root angiography.

Aortic valve regurgitation severity was classified as follows during angiography: None: no regurgitation observed. Mild: a small amount of contrast enters the left ventricle, clearing with each cardiac cycle. Moderate: contrast enters the left ventricle, discretely opacifying the entire left ventricle in diastole. Severe: complete, dense opacification of the entire left ventricle during the cardiac cycle.

In most patients, dilation was initiated with a balloon diameter of 90-100% of that of the aortic annulus, gradually increasing in size at the discretion of the operator until an adequate result was achieved. The procedure was considered successful if the immediate residual gradient after balloon dilatation was less than 35 mmHg and aortic valve insufficiency was less than or equal to moderate.

Follow-up 

All patients were followed at six-month intervals for at least two years. During follow-up visits, detailed echocardiographic assessments were performed, including measurements of left ventricular end-systolic and end-diastolic diameters, left ventricular wall thickness, left ventricular systolic function, aortic valve morphology, and continuous wave Doppler across the aortic valve. The outcome measure was defined as freedom from any reintervention (surgical repair or repeated balloon dilation) during the follow-up period. Surgical aortic valve repair included both aortic annuloplasty and valve replacement. Accordingly, patients were divided into two groups: those who underwent reintervention and those who did not.

Statistical analysis

The data were analyzed using SPSS version 25.0 (IBM Corp, Armonk, NY, USA). The Kolmogorov-Smirnov test was used to assess whether the data were homogeneously distributed. Student’s t-test was employed for comparing data from two independent groups if the data were homogeneous, while the Mann-Whitney U test was used for nonhomogeneous data. The chi-square test was utilized to compare group ratios. Statistical significance was considered for p-values less than 0.05.

## Results

Among the 48 patients who underwent balloon valvuloplasty due to aortic stenosis, 13 (27%) were female and 35 (73%) were male. The median age at the time of the procedure was 27.5 months (IQR: 4-96), the median weight was 9.9 kg (IQR: 5.40-29.50), and the median height was 79 cm (IQR: 54-133). The median follow-up duration was 93.5 months (IQR: 38-132) (Table 1). Echocardiographic measurements revealed a median peak gradient of 80 mmHg (IQR: 65-90) and a median mean gradient of 55 mmHg (IQR: 47-65) across the aortic valve. The median aortic valve annulus diameter was 10.5 mm (IQR: 8-12), the median left ventricular end-diastolic diameter was 2.85 cm (IQR: 2-3.6), and the median ejection fraction was 65% (IQR: 60-76). The bicuspid aortic valve was detected in 40 (83%) of the patients and the monocuspid valve was detected in 8 (17%) of them (Table 1). 

**Table 1:** Demographic and echocardiographic results of patients before BAV. LVEDD, left ventricular end-diastolic diameter; EF, ejection fraction; BAV, balloon aortic valvuloplasty

Characteristics	Results
Female/male, n (%)	13/35 (27/73)
Age, months (IQR)	27.5 (4-96)
Body weight, kg (IQR)	9.9 (5.40-29.50)
Height, cm (IQR)	79 (54-133)
Follow-up time, months (IQR)	93.5 (38-132)
Aortic valve morphology, n (%)	
Bicuspid	40 (83)
Monocuspid	8 (17)
LVEDD, cm (IQR)	2.85 (2-3.6)
EF% (IQR)	65 (60-76)
Mean aortic valve gradient, mmHg (IQR)	55 (47-65)
Aortic regurgitation, n (%)	
None	28 (58.3)
Mild-moderate	20 (41.7)
Severe	0

Angiographic assessments in all patients revealed a median left ventricular pressure of 160 mmHg (IQR: 140-200) and a median pressure gradient between the left ventricle and the aorta of 60 mmHg (IQR: 42-80). The median balloon diameter used was 10 mm (IQR: 8-12). Post-procedural measurements showed a median mean gradient of 30 mmHg (IQR: 20-35) between the left ventricle and the aorta (Table 2). 

**Table 2:** Angiographic measurements and follow-up results of the patients. ABV, aortic balloon valvuloplasty

Features	Results
Pre-ABV catheter peak systolic gradient, mmHg, (IQR)	60 (42-80)
Post-ABV catheter peak systolic gradient, mmHg, (IQR)	30 (20-35)
Pre-ABV aortic valve annulus, mm (IQR)	10.5 ( 8-12)
Balloon diameter, mm (IQR)	10 (8-12)
Balloon diameter/annulus diameter ratio (IQR)	0.96 (0.9-1.1)
Pre-ABV aortic regurgitation mild and moderate, n (%)	20 (41.6)
Post-ABV aortic regurgitation moderate and severe, n (%)	22 (45.8)
ABV success rate, n (%)	45 (93.7)
Aortic valve replacement, n (%)	11 (22.9)
Homograft, n (%)	1 (2.9)
Mechanical valve replacement, n (%)	5 (10)
Ross procedure, n (%)	5 (10)
Femoral artery occlusion, n (%)	2 (4.1)
Dysrhythmia, n (%)	5 (10.4)

Pre-procedural echocardiography revealed aortic insufficiency in 20 patients, with only one patient having moderate insufficiency and 19 patients having mild aortic valve insufficiency. Post-angiography left ventricular injection identified aortic insufficiency in a total of 22 patients, with two patients developing new insufficiency who previously had no aortic insufficiency. Among the seven patients who initially had mild insufficiency, the severity progressed to a moderate level. No patients exhibited severe aortic insufficiency.

When evaluated according to current guideline success criteria, the procedure was successful in 45 (93.75%) patients. Major complications during angiography included life-threatening arrhythmias in five patients: four developed ventricular tachycardia, and one developed ventricular fibrillation. The patient with ventricular fibrillation underwent CPR in the angiography room, reverted to sinus rhythm, and was subsequently transferred to the ICU. During follow-up, peripheral pulses were not palpable in two patients; Doppler ultrasound detected thrombosis in these two patients, which regressed after heparin infusion and low-molecular-weight heparin treatments. No deaths occurred during the angiographic procedure. 

Patients were divided into two groups based on follow-up: those managed without additional interventions and those who required aortic valve repair or replacement. Thirty-seven patients did not need further interventions during follow-up. Eleven patients required surgical treatment for aortic stenosis or insufficiency. Eleven patients underwent aortic valve surgery: five underwent the Ross procedure, one underwent homograft aortic valve replacement, and five underwent mechanical aortic valve replacement. The patients were evaluated for factors that could be predictive before aortic valve surgery. Patients were evaluated for surgical valve replacement according to their demographic, echocardiographic, angiographic, and follow-up echocardiographic parameters. Pre-procedural aortic insufficiency was identified as a risk factor for surgical intervention (p=0.024). No significant difference was detected between the two groups in other parameters. No death was detected in the early period after angiography, and only one patient died due to sepsis three months later.

## Discussion

Congenital aortic valve stenosis, even if initially mild, is a progressive condition that can lead to sudden death or left ventricular systolic dysfunction over time [8,9]. Historically, surgical valvotomy was the treatment of choice for congenital valvular aortic stenosis [10]. However, advancements in BAV have made it the first-line treatment, especially in neonates [11]. Both BAV and surgical valvotomy are palliative procedures aimed at delaying the need for aortic valve replacement until the patient reaches adulthood. The risk of restenosis varies between 15% and 65% across different centers [6]. Although BAV has a low immediate mortality risk, long-term outcomes include high rates of valve dysfunction and the need for reintervention, including aortic valve replacement. While BAV is safe and effective in relieving congenital aortic stenosis, it carries a long-term risk of residual aortic stenosis and progressive aortic insufficiency, necessitating valve replacement [12].

In our study, the majority of patients undergoing aortic balloon valvuloplasty were male, consistent with the higher prevalence of this condition in males, with a male-to-female ratio ranging from 3:1 to 5:1 [13]. Associated congenital heart diseases such as PDA, coarctation of the aorta, and ventricular septal defects are present in 15-20% of patients with valvular aortic stenosis. In patients with critical aortic stenosis, the presence of a PDA is expected to maintain systemic circulation [14]. The bicuspid aortic valve is the most frequent congenital anomaly and has a natural evolution toward aortic regurgitation or stenosis due to the asymmetrical valve function associated with an evolutive ascending aortopathy. The monocuspid aortic valve is a rare congenital cardiovascular anomaly, which is often misdiagnosed as a bicuspid aortic valve. These two valve structures are the most important causes of aortic stenosis. Our patient group was selected only as patients with isolated aortic stenosis and those who underwent percutaneous balloon valvuloplasty. Aortic valve valvotomy was performed simultaneously by our surgeons on patients who required other accompanying surgery.

In our study, we performed balloon valvuloplasty according to the established indications for aortic valvuloplasty in children with isolated valvar aortic stenosis. Current literature provides specific guidelines that we adhered to in determining the appropriate candidates for this procedure [6]. In our patient cohort, we identified similar findings via echocardiography and angiography, aligning with the criteria outlined in the literature. The procedure was successful in the majority of patients. In three cases after aortic balloon valvuloplasty, the intervention was considered unsuccessful because sufficient gradient reduction could not be achieved. Two of our unsuccessful patients had annulus hypoplasia along with valve stenosis, and therefore effective gradient reduction could not be achieved. It has been reported that ABV effectiveness is insufficient in cases with annulus hypoplasia. It has been shown in the literature that aortic hypoplasia increases failure and mortality during both surgical valvotomy and balloon valvuloplasty in the treatment of aortic stenosis.

In the long-term follow-up of the patients, it was determined that the degree of aortic insufficiency was higher in the group requiring surgical valve replacement. The incidence of significant aortic insufficiency has been reported as 2.2-29% in the literature [15,16]. These studies also showed that the rate of need for reintervention increased with the degree of aortic regurgitation. While there are studies showing that aortic regurgitation complication is more common in those who underwent valvuloplasty with a balloon larger than the diameter of the annulus (oversized), there are also studies that do not find a relationship [17,18]. In patients with pre-existing aortic insufficiency, the degree of insufficiency remained the same or increased slightly post-procedure. The reason why aortic insufficiency did not increase in our patient group may be due to the cautious selection of balloon diameter in the patients. The need for aortic valve replacement during follow-up was significantly higher in patients with aortic insufficiency compared to those without.

Complications associated with aortic balloon valvuloplasty include bleeding, thrombosis, infection, arrhythmias, and sudden cardiac arrest [19,20]. Complications related to aortic balloon valvuloplasty are more common in critical aortic stenosis of the newborn and aortic stenosis with severe LV systolic dysfunction. Among the major complications, only thrombosis and cardiac arrhythmia were detected in patients who underwent angiography. Malignant arrhythmias were detected in five patients during angiography. One patient underwent cardiopulmonary resuscitation due to ventricular fibrillation and the patient returned to sinus rhythm after the intervention. Femoral artery thrombosis, which developed in two patients, regressed with appropriate anticoagulant treatment. No deaths occurred during the angiographic procedure. It should not be forgotten how risky the BAV procedure is and the high mortality rate of major complications that may develop.

Both aortic balloon valvuloplasty and valvotomy are recognized as palliative treatment approaches. In line with the literature, the need for surgical valve replacement increased with longer follow-up periods in our patients [21]. The most commonly used procedures for patients requiring surgery after aortic balloon valvuloplasty were the Ross operation and mechanical aortic valve replacement, the latter being preferred in older patients [22]. In pediatric patients requiring aortic valve replacement, the Ross procedure is often preferred. This procedure involves replacing the diseased aortic valve with the patient's own pulmonary valve, which is then replaced with a donor pulmonary valve. The Ross procedure is advantageous because the autograft (the patient's own valve) can grow with the child, reducing the need for future replacements. Additionally, the pulmonary autograft tends to have better hemodynamic performance and lower thrombogenicity compared to mechanical valves. This approach minimizes the need for lifelong anticoagulation therapy, which is particularly beneficial for young patients [23].

The most important limitation of our study is that, as a feature of being a retrospective study, echocardiographic examinations were performed by different experts in the past and the valve structure, which may be difficult to decide in some cases, was evaluated subjectively. Additionally, since it was a retrospective study with a relatively small number of patients, prospective studies with a larger number of patients are needed.

## Conclusions

Our study reaffirms that BAV remains a crucial intervention for patients with congenital valvular aortic stenosis, particularly in the pediatric population. While BAV is effective in relieving stenosis and delaying the need for aortic valve replacement, it is essential to recognize its palliative nature and the associated long-term risks, such as residual aortic stenosis and progressive aortic insufficiency. Our findings underscore the importance of adhering to established guidelines to ensure optimal patient selection and outcomes. The presence of pre-existing aortic insufficiency significantly influences the long-term need for valve replacement, which is higher in these patients.
